# Soil enzymes activity: Effect of climate smart agriculture on rhizosphere and bulk soil under cereal based systems of north-west India

**DOI:** 10.1016/j.ejsobi.2021.103292

**Published:** 2021

**Authors:** H.S. Jat, Ashim Datta, Madhu Choudhary, P.C. Sharma, Bharti Dixit, M.L. Jat

**Affiliations:** a ICAR-Central Soil Salinity Research Institute (CSSRI), Karnal, Haryana, India; b International Maize and Wheat Improvement Center (CIMMYT), New Delhi, India; c Choudhary Charan Singh Haryana Agricultural University, Hisar, India

**Keywords:** Conservation agriculture, Climate smart agriculture, Rhizosphere, Rice-wheat system, Maize-wheat system, Long term managements

## Abstract

In agriculture production system, soil enzymes are important indicators of soil quality. Measurements of soil quality parameter changes are essential for assessing the impact of soil and crop management practices. Keeping this in view, an experiment was conducted to evaluate the enzyme activities namely dehydrogenase (DHA), β-glucosidase, acid and alkaline phosphatase (*AcP* & *AlP*), fluorescein diacetate hydrolases (FDH), cellulase, urease and aryl sulphatase in rhizosphere and bulk soil after 8 years of different management regimes. Soil organic carbon (SOC), moisture content and few enzyme indices such as enzymatic pH indicator (*AcP/AlP*), alteration index three (*Al3*) and geometric mean (*GMea*) were also measured. The treatments were conventional rice-wheat system (termed as scenario (Sc1), CT system), partial conservation agriculture (CA)-based rice-wheat-mungbean system (Sc2, PCA-RW), partial climate smart agriculture (CSA)-based rice-wheat-mungbean system (Sc3), partial CSA-based maize-wheat-mungbean system (Sc4), full CSA-based rice-wheat-mungbean system (Sc5), and full CSA-based maize-wheat-mungbean system (Sc6). Soil samples were collected from rhizosphere and away from roots (bulk soil) at 0–15 cm soil depth before sowing (from rhizosphere of previous crops), at maximum tillering, flowering, and after harvesting of wheat crop. Results showed that DHA activity was higher before sowing (59.8%), at maximum tillering (48.4%), flowering (8.6%) and after harvesting (19.1%) in rice based CSA systems (mean of Sc3 and Sc5) over maize based CSA systems (mean of Sc4 and Sc6) in rhizospheric soil. On average, β-glucosidase activity was significantly higher in rhizospheric soils of rice based system over maize based CSA system. Before sowing of wheat, significantly higher (21.4%) acid phosphatase activity was observed in rhizosphere over bulk soils of maize based CSA system. Significantly higher alkaline phosphatase activity was observed before sowing of wheat in bulk soils of rice (25.3%) and maize (38.5%) based CSA systems over rhizospheric soils. Rice based CSA systems showed 27% higher FDH activity than maize based systems. Significant interaction effect was observed between the managements and enzymes. SOC played an important role in regulating the enzymes activity both in rhizosphere and bulk soil. Significant variation in *AcP/AlP*, *Al3* and *GMea* was observed among the managements. Therefore, CSA managements are beneficial in improving enzyme activities not only in rhizosphere but also in bulk soil where residues are retained thereby may help in improving nutrient cycling.

## Highlights

• Soil enzyme activities in rhizosphere and bulk soil were carried out with climate smart agriculture practices.
• Residue retention and zero tillage improves enzyme activities in bulk soil.
• Dehydrogenase and β-glucosidase activities were higher in rhizospheric soils of rice based system.
• Urease activity was not affected by growth stages and climate smart agriculture practices.
• SOC played an important role in regulating the enzymes activity in soil.

## 1 Introduction

Soil enzymes are the key players in biochemical processes of organic matter recycling in the soil system and their activities are closely related to soil organic matter (SOM), soil physical properties, and microbial activity [1]. During decomposition of SOM and nutrient cycling, soil enzymes act as necessary catalysts and strongly influence energy transformation, environmental quality, and agronomic productivity. Soil enzymes provide early detection of changes in soil health because they respond to soil management changes and environmental factors much sooner than other soil quality parameters. Qualitative and quantitative changes in soil enzymes determine the availability of nutrients and crop productivity [2]. Different agricultural practices like tillage, cropping systems, irrigation and nutrient management influence soil enzyme activities, thereby influencing yield sustainability [3]. Adverse impacts of mechanical tillage, cropping systems, and residues removal have been observed in soil enzymatic activities and availability of plant nutrients [4]. Application of organic and inorganic fertilization exerts a strong influence on soil quality. In any agriculture production system, amending with organic matter and application of balanced fertilizers improve soil organic carbon and biological properties including microbial biomass and enzymatic activities [5,6]. Dehydrogenase enzyme activity (DHA) is considered as the indicator of oxidative activity of soil microorganisms and increases significantly upon application of balanced fertilization [6].

Climate Smart Agriculture (CSA) practices are based on conservation agriculture (CA) principles of zero tillage (ZT), residue management and sustainable crop rotation along with precision irrigation and N-management using sub-surface drip irrigation (SDI) system. There are number of advantages of CSA practices such as higher soil organic carbon, improved soil physical properties, nutrient availability, and crop productivity as reported by researchers all over the world [[7], [8], [9], [10]].

Several studies have been done on the effect of different agriculture management practices on soil enzyme activities in cereal based systems [[11], [12], [13]]. While studying the effect of series of CA based managements on soil enzymes, Choudhary et al. [14] reported 210% and 49% higher DHA and alkaline phosphatase activity (AlP), respectively in soils under maize-wheat-mungbean with residue retention and 140% and 42% under rice-wheat-mungbean system with residue retention over conventional rice-wheat system, respectively in NW India. Bergstrom et al. [12] compared six enzymes namely urease, glutaminase, phosphatase, arylsulfatase, β-glucosidase and dehydrogenase under no tillage along a topographic and soil textural gradient and observed higher β-glucosidase, glutaminase, phosphatase and aryl sulfatase activity in coarse-textured soils at a lower slope than in fine-textured soil at an upper slope. No-tilled soil showed higher dehydrogenase, urease, protease, phosphatase and β-glucosidase activities over conventional tillage system in sorghum cultivation under subtropical conditions [15].

The rhizosphere zone of the plants acts as hotspot of enzyme activities. Recently, it was stated that the rhizosphere activity should be extended from mm scale to cm scale due to H_2_ fertilization effect and volatile organic compounds released by roots [16]. The quantity and quality of root exudates depends on plant type and growth stages of plants [ 17] which also influence the diversity and activity of microbes, biochemical processes and enzyme activities [17]. ‘Rhizosphere priming effect’ is also an important factor playing an important role in SOM decomposition [17].

In most of the studies so far, soil samples were collected between rows of the crops after harvesting (called bulk soil sample). In conventional agricultural practices, tillage mixes the stubbles/roots of previous crop with soil before sowing of next crop and after planking stubbles are accumulated and removed from the field. But in CSA practices, stubbles and roots of the crops are undisturbed with zero tillage condition and loose crop residues are retained over the soil surface instead of burning or removal as practiced in conventional agriculture. Generally rhizosphere soil is characterized by higher microbial activity due to rhizodeposition, root secretion etc. than bulk soil (away from roots) [17]. Therefore, in CSA, we may expect higher enzymes activity in bulk soil compared to conventional practices due to residue retention and also enzymes activity in rhizosphere and bulk soil need to be investigated to capture whether there is any synergistic effect exists. There is hardly any study to unveil this aspect. The objectives of this study are to assess the enzyme activities and SOC concentration in rhizosphere and bulk soils and their interactions after 8 years of continuous smart crop management practices. We hypothesize that soil enzyme activities in bulk soil with CSA based management practices will be improved at different growth stages compared to those of conventional/tillage based management practices.

## 2 Materials and methods

### 2.1 Field experimental design

The experiment was established in 2009 at the research farm of Indian Council of Agricultural Research (ICAR) - Central Soil Salinity Research Institute (CSSRI) (29°70′N, 76°95′E), Karnal, India. Soil type is loam in texture with 34% sand, 46.1% silt and 19.9% clay. It falls under *Typic Natrustalf* category. Climate is extreme hot and dry (April–June) to wet summers (July–September) and cold dry winters (October–March). Average annual temperature is 26 °C with maximum and minimum of 34° and 18 °C, respectively with annual precipitation of 650 mm.

Initially, the experiment comprised of four cereal-based scenarios varying in cropping system, tillage, crop establishment methods, and residue management practices (Sc1, Sc2, Sc3 and Sc4). Treatments were replicated thrice in 20 m × 100 m plot size in randomized complete block design. In May 2016, precise water management practice (subsurface drip irrigation; SDI) was included in subdivided plots (20 m × 50 m) of Sc3 and Sc4, respectively. Briefly, six treatments termed as scenarios (Sc) were: i) conventional-till (CT) rice-CT wheat (Sc1; farmers' practice; CT); ii) CT rice-Zero tillage (ZT) wheat-ZT mungbean with flood irrigation (Sc2; partial CA); iii) ZT rice-ZT wheat-ZT mungbean with flood irrigation (Sc3; rice based partial CSA); iv) ZT maize-ZT wheat-ZT mungbean with flood irrigation (Sc4; maize based partial CSA); v) ZT rice-ZT wheat-ZT mungbean with SDI (Sc5; rice based full CSA); and vi) ZT maize-ZT wheat-ZT mungbean with SDI (Sc6; maize based full CSA). Sc3 and Sc4 were based on principles of CA practices where irrigation water and N application were not precisely managed and called it partial climate smart agriculture (CSA). However, in Sc5 and Sc6, irrigation water and N in the form of urea was precisely applied using subsurface drip irrigation (SDI) and called full CSA. Best crop management practices were followed in all the treatments except Sc1, where farmer's traditional practices were followed (Supp. Table 1, Supp. Fig. 1). We used four systems for convenience, conventional tillage based rice-wheat system (CT-RW), partial CA based rice-wheat mungbean system (PCA-RW) (Sc2), rice based CSA system (mean of Sc3 and Sc5) and maize based CSA system (mean of Sc4 and Sc6).

### 2.2 Soil sampling, processing and analysis

Wheat is common crop among all the scenarios so soil samples were collected from wheat season in order to explore the effect of management practices (CT v/s PCA and/CSA) on different enzyme activities like dehydrogenase (DHA), acid phosphatase (AcP), alkaline phosphatase (AlP), beta-glucosidase (β-glu), fluorescein diacetate hydrolases (FDH), aryl sulphatase (ArS), urease (Ur) and cellulose (CeL) activity and SOC changes. From each plot, soil samples were collected at 0–15 cm soil depth by an auger from nine locations from each rhizosphere and non-rhizosphere zones (bulk soils) and composite samples were prepared separately before sowing, maximum tillering, flowering, and after harvesting in the year 2017–18 (wheat was sown in November 2017 and harvested in April 2018). For rhizosphere zone samples, wheat plants were uprooted and soil adhered to roots was collected. Bulk soil samples were collected from the mid-point of the two rows. Row to row distance in wheat crop was 22.5 cm. Rhizosphere soil before sowing indicates rhizosphere of previous crops. As after harvesting of previous crop (rice and maize), the stubbles remain intact in soil, we collected soil samples from the rhizosphere of those crop stubbles and designated as rhizosphere soil before sowing. Fresh soil samples were immediately stored in a refrigerator at 4 °C till analysis of different enzymes. DHA, AcP and AlP activities were estimated as described by Dick et al. [18]. β-glucosidase activity was determined by the method of Eivazi and Tabatabai [19], urease was by the method of Tabatabai [20] and aryl sulfatase was by the method of Tabatabai and Bremner [21]. Cellulase activity was measured by the method of Hope and Burns [22] and FDH assay by the method of Green et al. [23].

Root mass after harvesting of rice, wheat and maize was measured by using standard procedure. Soil blocks up to 40 cm depth with plant roots were taken out from four random places in each scenario. Roots were washed carefully, detached from the main stem at the first node. Roots were dried at 65 ± 5 °C at oven and dry weight was calculated and then converted to t/ha.

Soil moisture content was determined by drying the fresh soil samples at 105 °C for 24 h in a hot air oven until a constant weight and calculated by following formula.

Moisture content (%) = (Moist soil wt-dry soil wt)/dry soil wt × 100.

One part of the fresh samples collected from both rhizosphere and bulk soil was dried in shade, ground and sieved and stored in plastic container for chemical analysis. Oxidizable organic carbon (SOC) was determined by following Walkley and Black method [24].

The enzymatic pH indicator was calculated using the results of alkaline and acid phosphatase activity [25]:

Enzymatic pH indicator = *AlP/AcP*

The alteration index three (*Al3*) was also calculated using the results of β-glucosidase, phosphatase and urease [26]:

Alteration index three (*Al3*) = 7.87 β-glucosidase−8.22 acid phosphatase−0.49 urease

For each scenario, the geometric mean (*GMea*) was calculated as the mean for the assayed enzymes activities [27]. It is a general index to consolidate information from variables with different units and range of variation:

*GMea* = (DHA * GLU * AlP *AcP * FDH* Ur*CeL*ArS) ^1/8^

Where DHA, GLU, AlP, AcP, FDH, Ur, CeL, ArS are dehydrogenase, β-glucosidase, alkaline phosphatase, acid phosphatase, fluorescein diacetate hydrolases, urease, cellulase and aryl sulphatase, respectively.

### 2.3 Residue load

Crop residues recycled in each year under different scenarios are presented at Supp. Table 2. Significantly higher residues amount (129 Mg ha^−1^) were recycled in maize based system (mean of Sc4 and Sc6) over others, followed by PCA-RW (Sc2) (115.5 Mg ha^−1^). About 111 Mg ha^−1^residues were added in rice based CSA systems (mean of Sc3 and Sc5) during the last 8 years.

### 2.4 Statistical analysis

The data were subjected to analysis of variance (ANOVA) and using the general linear model procedure of the SPSS window version 17.0 (SPSS Inc., Chicago, USA). Treatment means were separated by Duncan Multiple Range Test (DMRT) at 5% level of significance (*P* < 0.05). Correlation study was performed among the enzymes, indices calculated from different enzymes, residue load, SOC and soil moisture content. To determine the effect of scenarios, rhizosphere/bulk soils and stages (fixed factors) and their interaction effect on the different enzyme activities (random variable), three-way ANOVA was carried out. Linear contrasts were used to compare single or multiple treatments against one another.

## 3 Results

### 3.1 Dehydrogenase (DHA) and β-glucosidase activity as influenced by management practices

**Fig. 1:**
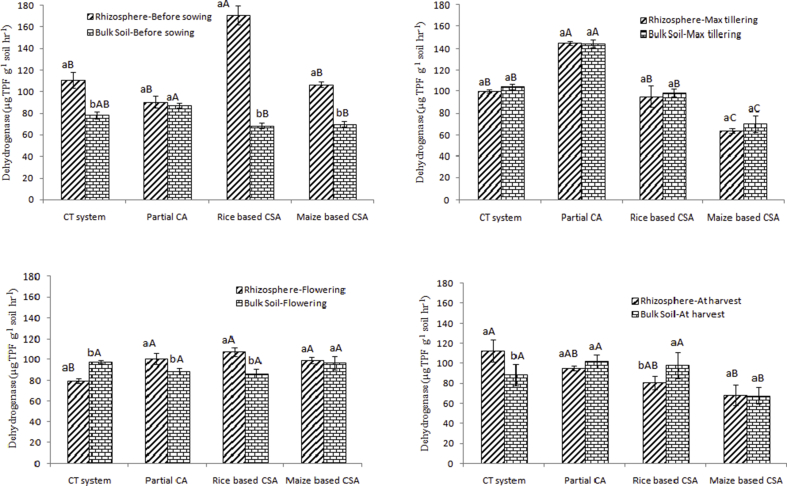
Dehydrogenase activity (μg TPF g^−1^ soil hr^−1^) in rhizosphere and bulk soils a) before sowing of crop, b) at maximum tillering c) flowering stage of crop and d) after harvesting of crop under different tillage, residue and crop rotations. Same upper case letters among the CSA systems and same lower case letters between rhizosphere and bulk soils in each system are not significantly different at P < 0.05 according to Duncan Multiple Range Test (DMRT) for separation of mean.

Significant variation in DHA was observed both in rhizosphere and bulk soils of different scenarios (Fig. 1). Before sowing of the crop, DHA activity was significantly higher in rhizospheric soils over bulk soils irrespective of cropping system. In rhizospheric soil, DHA activity was significantly higher before sowing (59.6%) and flowering stage (18.7%) in rice based CSA systems (mean of Sc3 and Sc5) over maize based CSA systems (mean of Sc4 and Sc6) (Fig. 1a and 1c). Before sowing in maize based system, about 35% higher DHA activity was observed at rhizospheric soil over bulk soil. At maximum tillering stage, DHA activity was significantly higher in partial CA based rice system (PCA-RW, Sc2) (145 μg TPF g^−1^ soil hr^−1^) over others irrespective of sampling location (Fig. 1b). At flowering, DHA activity was 12% higher in rhizosphere of PCA-RW (Sc2) over bulk soil (Fig. 1c). After harvesting of the crop, significantly higher (21%) DHA activity was observed in bulk soils (98 μg TPF g^−1^ soil hr^−1^) over rhizospheric soils (81 μg TPF g^−1^ soil hr^−1^) in rice based CSA system (Fig. 1d). Being the responsible enzyme for carbon cycle in soil, on average β-glucosidase activity was significantly (p < 0.05) higher in rhizospheric soils of rice based CSA systems (108 μg p-NP g^−1^ soil hr^−1^) over bulk soil (92 μg p-NP g^−1^ soil hr^−1^) whereas under maize based systems similar values were observed (Suppl. Fig. 2) irrespective of crop growth stages. In bulk soil under maize systems significantly higher β-glucosidase activity was observed before sowing (Suppl. Fig. 2a) and at maximum tillering stage (Suppl. Fig. 2b) compared to rice based CSA system. Whereas at harvesting stage, rhizospheric soils under maize systems (112 μg p-NP g^−1^ soil hr^−1^) showed significantly (p < 0.05) higher β-glucosidase activity over rhizospheric soil under rice based CSA system (94 μg p-NP g^−1^ soil hr^−1^) (Suppl. Fig. 2d). At maximum tillering stage, significantly higher β-glucosidase activity was observed at rhizosphere soil of rice based CSA system (93 μg p-NP g^−1^ soil hr^−1^) over bulk soil (72 μg p-NP g^−1^ soil hr^−1^) and also maize based system (78 μg p-NP g^−1^ soil hr^−1^) (Suppl. Fig. 2b). In rice based CSA system, after harvesting of the crop significantly (p < 0.05) higher β-glucosidase activity was observed in bulk soils (12.8%) over rhizospheric soils (Suppl. Fig. 2d). On average β-glucosidase activity was 20% higher (p < 0.05) in CA based scenarios (Sc2-Sc6) over conventional tillage scenario (Sc1) irrespective of crop growth stages and sampling location.

### 3.2 Acid and alkaline phosphatase activity under different managements

Significant variation in acid and alkaline phosphatase activity was observed irrespective of scenarios and sampling location (Supp. Table 3 and 4). Before sowing of wheat, significantly higher (21.4%) acid phosphatase activity was observed in rhizosphere over bulk soils of maize based systems (Supp. Table 3). But at maximum tillering, about 8.2% higher acid phosphatase activity was observed in bulk soils over rhizosphere soil of rice based CSA systems. In maize based systems, at flowering stage 6% higher (p < 0.05) acid phosphatase activity was recorded at bulk soil over rhizospheric soil. At harvesting, bulk soils recorded significantly higher acid phosphatase activity in both rice (9.5%) and maize (7.4%) based CSA systems over rhizospheric soils (Supp. Table 3). Significantly higher alkaline phosphatase activity was observed before sowing of wheat in bulk soils of rice (25.3%) and maize (38.5%) based CSA systems over rhizospheric soils (Supp. Table 4).

### 3.3 Fluorescein diacetate hydrolases (FDH) and aryl sulphatase (ArS) activity under different managements

**Fig. 2:**
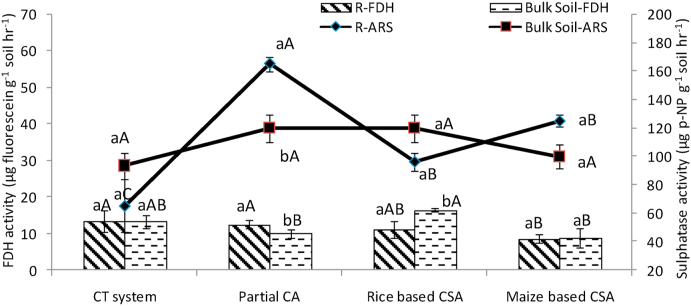
Fluorescein diacetate hydrolases (FDH) (μg fluorescein g^−1^ soil hr^−1^) and Aryal sulphatase activity (μg p-NP g^−1^ soil hr^−1^) in rhizosphere and bulk soils at maximum tillering stage of crop under different tillage, residue and crop rotations. *Where*, R: rhizosphere; ARS: aryal sulphatase activity; FDH: Fluorescein diacetate hydrolases activity. Same upper case letters among the CSA systems and same lower case letters between rhizosphere and bulk soils in each system are not significantly different at P < 0.05 according to Duncan Multiple Range Test (DMRT) for separation of mean.

**Fig. 3:**
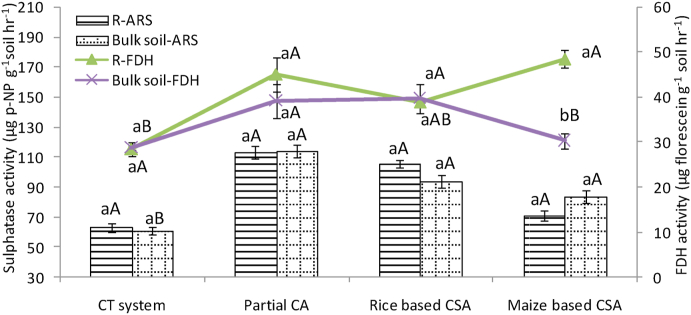
Fluorescein diacetate hydrolases (μg fluorescein g^−1^ soil hr^−1^) and Aryal Sulphatase activity (μg p-NP g^−1^ soil hr^−1^) in rhizosphere and bulk soils at flowering stage of crop under different tillage, residue and crop rotations. *Where*, R: rhizosphere; ARS: aryal sulphatase activity; FDH: Fluorescein diacetate hydrolases activity. Same upper case letters among the CSA systems and same lower case letters between rhizosphere and bulk soils in each system are not significantly different at P < 0.05 according to Duncan Multiple Range Test (DMRT) for separation of mean.

On average rice based CSA system showed 27% higher FDH activity than maize based system (Supp. Table 5). In rhizosphere, on average it was 18% and in bulk soils it was 38% higher in rice based CSA systems than maize based systems. At maximum tillering stage, about 29% higher FDH activity was observed in bulk soils of rice based CSA system over rhizosphere soil whereas rhizospheric soils of partial CA based system recorded 11.5% higher FDH activity over bulk soils (Fig. 2). Rhizosphere soils under maize based systems showed about 39% higher FDH activity over bulk soils at flowering stage (Fig. 3). Similar FDH activity was observed under partial CA and rice based CSA systems in rhizosphere and bulk soils (Fig. 3). After harvesting, bulk soils under maize based CSA and partial CA based systems recorded 29% and 69% higher FDH activity over rhizosphere soils (Suppl. Fig. 3). In rhizosphere zone an increase of 14% FDH activity can be seen in CA based systems (mean of Sc2 to Sc6) over CT system (Sc1) but simultaneously 6% decrease was noticed in CA based systems over CT system in bulk soils (Supp. Table 5).

At different growth stages of wheat crop, variation in activities of ArS (17.63–117.58 μg p-NP g^−1^soil hr^−1^) was recorded for rhizosphere and bulk soils (Supp. Table 6). In both rhizosphere and bulk soil, ArS activity was higher by 12% and 15%, respectively before the sowing of wheat than the tillering stage. It was first decreased from sowing to tillering and then increased from tillering to flowering in both soils. At maximum tillering stage, about 32% higher aryl sulphatase activity was observed in rhizosphere soils of partial CA based system (Sc2) over bulk soil (Fig. 2). Highest activity of ArS was recorded at flowering stage with few exceptions (Fig. 3). It was noticed that lowest activities of ArS was found in conventional till scenario (CT system, Sc1) irrespective of growth stages and sampling location. In both the zones, on average, 28% higher ArS activity was recorded in PCA and CSA based scenarios (mean of Sc2 to Sc6) over CT system (Sc1). Overall activities of ArS were found similar in all scenarios in both rhizosphere and bulk soils, with a mean value of 60 μg p-NP g^−1^soil hr^−1^ in both the zones. Rice based partial CSA system (Sc3) has more ArS activities than maize based partial CSA (Sc4) except in rhizosphere soil of maximum tillering stage. In rhizosphere and bulk soil, it was 13% and 12% higher in rice based CSA system than maize based system, respectively (Supp. Table 6).

### 3.4 Urease (ur) and cellulase (CeL) activity under different managements

Higher activity of urease was noticed in maize based full CSA system (Sc6) compared to other scenarios (Supp. Table 7). There was hardly any effect of growth stages on Ur in both rhizosphere and bulk soils. Effect of CSA practices has not been observed on Ur activities as it was found similar in CA based scenarios (mean of Sc2 to Sc6) (319.9 μg urea g^−1^ soil hr^−1^) and CT system (319.6 μg urea g^−1^ soil hr^−1^) (Supp. Table 7).

Cellulase activity was found to increase from before sowing to maximum tillering and flowering stage with some exceptions and decreased towards harvesting stage (Supp. Table 8). In CA based scenarios (mean of Sc2 to Sc6) significantly higher CeL activities (15.54 μg glucose g^−1^ soil hr^−1^) were noticed over CT system (8.03 μg glucose g^−1^ soil hr^−1^). Partial CA system (Sc2) showed 52% increase in CeL activities over CT system. Integration of mungbean (mean of Sc3 to Sc6) showed 34% increase in CeL activity over partial CA.

### 3.5 Interactions effect of managements, sampling locations and crop growth stages on soil enzymes

The analysis of variance showed that all the enzymes in rhizosphere and bulk soils were significantly influenced by scenarios, rhizosphere (R)/bulk soil (B), crop growth stages and their interactions i.e. scenario × stage, scenario × R/B, stage × R/B and scenario × stage × R/B except few instances (Table 1). The interaction effect between rhizosphere × bulk soils was not significant for acid and alkaline phosphatase activity whereas crop growth stage × R/B was also not significant for acid phosphatase activity. Contrast analysis showed that there was significant difference between rice and maize based cropping systems (Table 1).

**Table 1:** Interactions among the scenarios, crop growth stages and rhizosphere and bulk soil and contrast effect between rice-wheat and maize - wheat systems.

	Statistical significance (P value)							
Source of variation	DHA	AcP	AlP	ArS	Ur	β-Glu	FDH	CeL
Scenario	<0.0001	0.0174	<0.0001	<0.0001	0.0001	<0.0001	0.0105	<0.0001
Stage	<0.0243	<0.0001	<0.0001	<0.0001	<0.0003	<0.0001	<0.0001	<0.0001
Scenario*stage	<0.0001	<0.0001	0.0201	<0.0001	<0.0001	<0.0001	<0.0001	<0.0001
Rhizosphere/bulk soil	<0.0001	0.4524	0.4524	<0.0001	<0.0001	<0.0001	<0.0002	<0.0001
Scenario*R/B	<0.0001	0.0324	0.0008	<0.0001	0.0020	<0.0001	0.0112	<0.0004
Stage*R/B	<0.0001	0.2097	<0.0001	<0.0001	<0.0001	<0.0001	<0.0001	<0.0001
Scenario*stage*R/B	<0.0001	0.0297	<0.0001	<0.0001	<0.0001	<0.0001	<0.0001	<0.0001
Contrast – Rice-wheat: Maize-Wheat	0.001	0.001	0.001	0.001	0.001	0.001	0.001	0.001

Where R: rhizosphere; B: bulk soil.

DHA: Dehydrogenase, AcP: Acid Phosphatase, AlP: Alkaline Phosphatase, ArS: Arylsulfatase.

Ur: Urease, β-Glu: β-glucosidase, FDH: Fluorescein diacetate hydrolases, CeL: Cellulase.

### 3.6 Soil organic carbon and soil moisture under different managements and crop growth stages

**Fig. 4:**
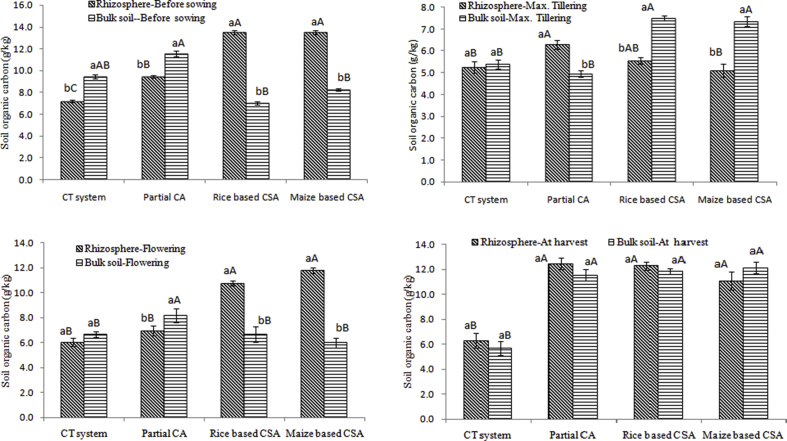
Soil organic carbon (g/kg) in rhizosphere and bulk soils a) before sowing of crop, b) at maximum tillering c) flowering stage of crop and d) after harvesting of crop under different tillage, residue and crop rotations. Same upper case letters among the CSA systems and same lower case letters between rhizosphere and bulk soils in each system are not significantly different at P < 0.05 according to Duncan Multiple Range Test (DMRT) for separation of mean.

Significant variation in SOC was observed at different crop growth stages and sampling locations. On average irrespective of crop growth stages and sampling locations, rice and maize based CSA (45%) and PCA-RW (37%) recorded significantly higher SOC over conventional practices (Fig. 4). Before sowing, rhizosphere soils of CSA based rice and maize systems recorded 90% (in rice rhizosphere) and 63% (in maize rhizosphere) higher SOC over their respective bulk soils. Whereas 18–24% higher SOC was observed in bulk soils under CT (rice crop) and PCA-RW system (rice crop) over rhizosphere soils (Fig. 4a). At maximum tillering stage, significantly higher SOC was observed in bulk soils under CSA based rice (36%) and maize (44%) based system over rhizosphere soils but about 21.4% lower SOC was observed in bulk soils over rhizosphere soil under PCA-RW system (Fig. 4b). At flowering stage, higher SOC concentration was observed in rhizosphere soils compared to maximum tillering stage irrespective of scenarios (Fig. 4c). But in bulk soils, significantly lower SOC were observed in rice (11%) and maize (18%) based system whereas PCA-RW system recorded 66% higher SOC compared to the bulk soil at maximum tillering stage (Fig. 4c). At harvesting stage, higher SOC was observed in all the scenarios irrespective of sampling locations except the bulk soil under CT system which registered 14% lower SOC compared to the bulk soil under flowering stage (Fig. 4d).

Residue retention exhibited strong influence on soil moisture content in all the scenarios (Supp. Table 9). At maximum tillering stage, similar moisture content was observed irrespective of scenarios. CSA based rice system recorded 34% higher moisture content at rhizosphere soil over bulk soil whereas 51% higher moisture was observed at bulk soils under maize based system over rhizosphere soil at flowering stage. On average 64% higher moisture content was observed at bulk soils under CSA based rice and maize based system over rhizosphere soil (Supp. Table 9).

### 3.7 Enzymatic pH indicator, *Al3* and *GMea* and their relationship with enzymes, residue load, SOC and soil moisture

**Fig. 5:**
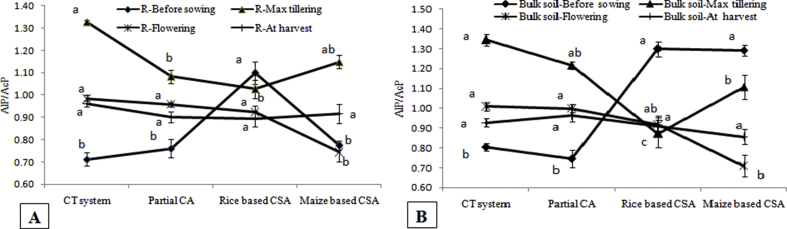
Enzymatic pH indicator (AlP/AcP) in A) rhizosphere and B) bulk soils before sowing of crop, maximum tillering, flowering stage and after harvesting of crop under different tillage, residue and crop rotations. Same lower case letters among the management system are not significantly different at P < 0.05 according to Duncan Multiple Range Test (DMRT) for separation of mean.

**Fig. 6:**
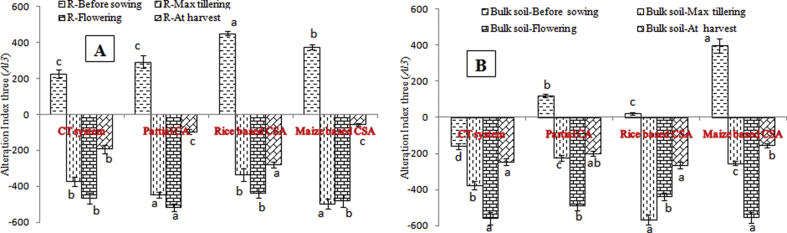
Alteration index three (Al3) in A) rhizosphere and B) bulk soils before sowing of crop, maximum tillering, flowering stage and after harvesting of crop under different tillage, residue and crop rotations. Same lower case letters among the management system are not significantly different at P < 0.05 according to Duncan Multiple Range Test (DMRT) for separation of mean.

**Fig. 7:**
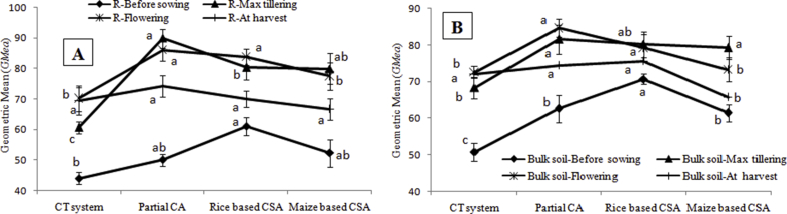
Geometric mean (*GMea*) in A) rhizosphere and B) bulk soils before sowing of crop, maximum tillering, flowering stage and after harvesting of crop under different tillage, residue and crop rotations. Same lower case letters among the management system are not significantly different at P < 0.05 according to Duncan Multiple Range Test (DMRT) for separation of mean.

The enzymatic pH indicator was calculated using the values of the alkaline and acid phosphatase activities under different managements. The value of this indicator varied from 0.71 to 1.34 irrespective of sampling location and crop growth stages (Fig. 5). Enzymatic pH indicator was significantly negatively correlated with β-glucosidase activity (r = −0.95, p < 0.05), *Al3* (r = −0.97, p < 0.05), SOC (r = −0.79, p < 0.05) and residue load (r = −0.82, p < 0.05) irrespective of sampling location and crop growth stages (Table 2). Alteration index three varied significantly among the crop growth stages and rhizosphere and bulk soil under different managements (Fig. 6). Lower values of *Al3* indicated better soil quality. In rhizosphere soil, lowest *Al3* (−516) was observed at flowering stage of partial CA based system whereas in bulk soil rice based CSA system recorded lowest *Al3* (−567) at maximum tillering stage. Significantly higher *Al3* values were recorded before sowing of crop irrespective of sampling location (Fig. 6). Significant negative correlation was observed between *Al3* and β-glucosidase (r = −0.94, p < 0.05), and also negatively correlated with residue load (r = −0.84, p < 0.05) and SOC (r = −0.73, p < 0.05) (Table 2). Significant variation in *GMea* index values was recorded among the managements and sampling locations. In rhizosphere and bulk soil, highest *GMea* index was observed in maximum tillering (90) and flowering stage (85) of partial CA based system (Fig. 7). Lower values of *GMea* index was recorded in conventional system and before sowing of wheat irrespective of sampling location. Significant positive correlation was observed between *GMea* and β-glucosidase (r = 0.73, p < 0.05), acid phosphatase (r = 0.99, p < 0.05), aryl sulphatase (r = 0.95, p < 0.05), SOC (r = 0.93, p < 0.05) and residue load (r = 0.84, p < 0.05) (Table 2). CA based management practices recorded significantly higher soil moisture over conventional system and significantly positively correlated with acid phosphatase (r = 0.84, p < 0.05), aryl sulphatase (r = 0.70, p < 0.05), cellulase (r = 0.86, p < 0.05), *GMea* (r = 0.88, p < 0.05), residue load (r = 0.84, p < 0.05) and SOC (r = 0.80, p < 0.05) irrespective of managements and sampling location (Table 2).

**Table 2:** Pearson's bivariate correlations among different enzymes and enzyme indices, SOC, residue load, moisture content irrespective of scenarios, crop growth stages and sampling locations.

Correlations														
	β-Glu	AcP	AlP	DHA	ArS	Ur	FDH	CeL	AL	EP	GM	SOC	RL	MC
β-Glu	1													
AcP	0.81*													
AlP	−0.09	0.47												
DHA	−0.19	0.35	0.98*											
ArS	0.69	0.96*	0.66	0.56										
Ur	0.38	−0.13	−0.56	−0.48	−0.13									
FDH	−0.09	0.50	0.97*	0.93*	0.64	−0.71								
Cl	0.77	0.64	−0.26	−0.42	0.40	−0.07	−0.12							
AL	−0.94*	0.56	−0.42	−0.49	0.40	0.61	−0.43	0.72						
EP	−0.95*	−0.62	0.26	0.31	−0.52	−0.65	0.31	−0.59	−0.97*					
GM	0.73*	0.99*	0.53	0.41	0.95*	−0.27	0.59	0.63	0.45	−0.51				
SOC	0.92*	0.97*	0.27	0.16	0.90*	0.07	0.29	0.71*	−0.73*	−0.78*	0.93*			
RL	0.96*	0.88*	0.06	−0.13	0.74*	0.11	0.06	0.88*	−0.84*	−0.82*	0.84*	0.95*		
MC	0.66	0.84*	0.26	0.09	0.70*	−0.44	0.40	0.86*	0.44	−0.38	0.88*	0.80*	0.84*	1

*. Correlation is significant at the 0.05 level (2-tailed).

Where β-Glu: β-glucosidase, AcP: Acid Phosphatase, AlP: Alkaline Phosphatase, DHA: Dehydrogenase, ArS: Arylsulfatase, Ur: Urease, FDH: Fluorescein diacetate hydrolases, CeL: Cellulase, Al: alteration index three, EP: enzymatic pH indicator, GM: geometric mean, SOC: soil organic carbon, RL: residue load, MC: moisture content.

## 4 Discussion

Climate smart agriculture management influences soil enzyme activities at different extents. Significant variations were observed for activities of the enzymes studied under partial CA, CSA and CT practices. Significant variation among the enzymes in rhizosphere and bulk soils among the scenarios at different crop growth stages might be due to the crop and soil management practices followed. Generally the activity of the microorganisms are higher at rhizosphere zone of the crop because it has been found that the proportions of rhizodeposition carbon (C) of below ground carbon inputs through roots, rhizodeposition etc. averaged 54–63% for the cereals [28]. Because of this labile carbon, the activity of the microorganisms at rhizosphere is higher compared to bulk soil resulting in higher enzymes activity in rhizosphere. Moreover, zero tillage, resource (irrigation water and nutrients) management and suitable crop rotation with mungbean integration facilitated congenial environment for the microorganisms.

Higher DHA activity in bulk soils after harvest of the rice crop might be due to the availability of very labile carbon originated from decomposition of previous year's wheat and mungbean residues. Decomposition of earlier surface retained maize residues releases labile carbon which was available to microbes and resulted in higher DHA activity in bulk soils under maize based system than rice based CSA system at flowering stage. Lower C: N ratio of maize roots (35:1) and stover (57:1) over rice (root: 58.8 and straw: 67:1) facilitated faster decomposition of maize residues. Higher root biomass of maize (3.8 t ha^−1^) over rice root mass (2.26 t ha^−1^) had resulted higher root carbon input to soil under maize system. Bergstrom et al. [12] reported higher DHA activity under zero tillage conditions. In a lab experiment Datta et al. [29] also showed faster decomposition of maize residues placed at surface soil in respect to maize as well as rice and wheat residues and their mixtures incorporated and placed at soil surface. Similar or higher activities of enzymes in bulk soil over rhizosphere soil was attributed to the production of volatile organic compounds released by roots which can be carried far away from rhizosphere in dry soil due to higher air filled porosity resulting in higher microbial activity [16]. ‘Rhizosphere priming effect’ mediated by microorganisms also plays an important role in increasing and decreasing enzymes activity in rhizosphere and bulk soil [17]. Higher β-glucosidase activity in rhizospheric soils over bulk soil under rice based CSA systems might be due to higher carbon input from fibrous root mass of rice in previous year (Suppl. Fig. 2a). But higher β-glucosidase activity in bulk soils under maize based systems at maximum tillering stage might be due to the higher residue load from maize residue (Suppl. Fig. 2b) leading to more carbon input to soil. This is also supported by the higher soil carbon concentration under maize based systems over rice based CSA systems (Fig. 3) and significant positive correlations between β-glucosidase activity with residue load and SOC (Table 2). Moreover, during flowering stage the decomposition of the previous year's residue were at peak as revealed from temperature rise at the end of February which might facilitate higher β-glucosidase activity at maize based systems. The flowering in wheat starts around mid-February and that time the temperature (22.8 °C) and relative humidity (73%) is conducive for microbial growth compared to January (18.4 °C and 79.5%). CA based scenarios received higher carbon input in the form of residue (Table 2) and zero tillage practice which resulted in the higher activity of β-glucosidase in soil. Pausch and Kuzyakov [30] reported that the highly dynamic nature of rhizodeposition and rhizodeposits are rapidly incorporated into microorganisms, soil organic matter, and decomposed to CO_2_ which explains the higher β-glucosidase activity in rhizospheric soil under rice. Before sowing of wheat, higher root biomass of maize (3.8 t ha^−1^) in addition of previous years residues left in the soil which is decomposing and supplying labile carbon to microbes, led to higher acid phosphatase activity. Hirte et al. (2018) [31] showed higher root biomass of maize (186 ± 15 g m^−2^) over wheat root biomass (137 ± 6 g m^−2^) after harvesting in long term field trial of DOK (bio-Dynamic, bio-Organic, Conventional site in Switzerland). Higher rhizodeposition in root zone led to the acidity which further accentuates the acid phosphatase activity [30]. Higher acid phosphatase activity in bulk soils at maximum tillering stage of rice and flowering stage of maize system is attributed to differential residue decomposition leading to varying rates of labile carbon release in these systems. After harvesting in both the system, the residues placed away from root zone were completely decomposed whereas the roots just start decaying which explains higher acid phosphatase activity in bulk soils. Similar observations were also reported in alkaline phosphatase activity. Crop rotation particularly inclusion of legume has played an important role in enzyme activities observed in those CSA based scenarios. In our case, legume integration in rice-wheat and maize-wheat systems has facilitated higher microbial activity leading to release of both acid and alkaline phosphatase activity in soil. Plant roots release a wide range of compounds that may differ between plant species [30] which leads to the difference between FDH activity in rice and maize based systems. Different crop rotation is also a factor in variation of FDH activities [32]. Different plant species results distinct microbial communities with different activity [33]. CSA practices have positive effect on soil enzymatic activities [14] and this can be seen in the overall FDH activity in CSA based scenarios and particularly in rhizosphere zone. Sulphatases are reported to be also found as exoenzymes in the soil and are closely linked to organic matter [34] applied in the form of heavy load of residues. Higher SOC in PCA and CSA based scenarios (Sc2 to Sc6) (Fig. 4) resulted in higher activities of ArS as evidenced from significant positive correlations between SOC and ArS (Table 2). Kotkova et al. [34] reported that in wheat ArS activity was higher in rhizosphere as compared to bulk soil and vice versa was reported in lupine but in our study such type of trend was not reported. In this study both the zones have similar activities of ArS. Higher ArS activity in rice over maize based CSA can be linked to differences in crop rotation and their residues as rice and maize were grown before wheat in those scenarios. Activity of cellulase at different growth stages can be regulated by the available moisture content due to crop residue mulch and irrigation water given to crops as evidenced from Supp. Table 9.

Higher β-glucosidase and acid phosphatase activity in no till soil over conventional agriculture in varying textured soils were also reported by other researchers [12,35,36]. Choudhary et al. [11,14] also reported higher DHA and alkaline phosphatase activity under zero till based conservation agriculture practices. Residue retention and zero tillage are the main reasons behind the higher CeL activity under CSA based scenarios. Residues provide favourable conditions to the microbes and microbial transformation of crop residue and soil organic matter enhances enzyme activities under zero tillage systems [8]. Higher enzyme activities in CSA based scenarios (both rice and maize based systems) as compared to CT and partial CA was mainly due to the integration of legume [14].

Higher SOC under CSA and PCA based rice and maize systems was due to higher residue load (112–129 Mg ha^−1^) (Suppl. Table 2) which supplies organic carbon to soils in addition to carbon input from plants through roots, rhizodepositions, secretions etc. We do not have measurements on plant and root derived carbons. Generally rhizosphere soil is characterized by higher amount of very labile carbon and lower contents of mineral nitrogen as well as other nutrients with 19–32 times higher number of microorganisms compared to bulk soil [17]. In bulk soil (away from roots), all the nutrients are mostly available with limiting easily available carbon for microbial growth [17]. Priming effect plays an important role in increasing or decreasing soil organic matter decomposition in rhizosphere as well as bulk soil with crop residue retention at soil surface under CSA [17,37]. Before sowing of wheat, lower SOC in maize rhizosphere soil compared to rice might be due to faster decomposition of maize roots with lower C:N ratio (35:1) over rice roots (C:N ratio of 58.8:1) thereby facilitating positive priming effect. Also maize being a C4 plant releases less organic compounds to soil through roots due to lesser investment of C in the below ground processes over C3 rice crop [17]. As the stubbles of rice and wheat were kept and mixed properly with soil during puddling under CT and PCA system resulting higher SOC in bulk soil compared to rhizospheric soil of wheat which might have experienced positive priming leading to lower SOC. At maximum tillering stage, lower SOC in rhizosphere soil compared to bulk soil in CSA based rice and maize systems might be due to higher rhizosphere priming effect caused by root secretions, rhizodeposition with vigorous microbial activity. In bulk soil at flowering stage, lower SOC was observed under rice and maize based CSA systems which might be due to higher priming effect mediated by greater activity of microorganisms caused by decomposing residues retained at soil surface. Whereas at rhizosphere soil, there might be higher rhizodeposition which results in higher SOC but with limiting mineral nitrogen leads to lower decomposition [17]. Significantly higher SOC in bulk soil at flowering under PCA system might be due to higher humification of crop residue carbon to SOC caused by proper mixing of crop residues with soil during previous puddled rice crop. At harvesting stage except CT system, higher SOC both at rhizosphere and bulk soil might be due to higher carbon released by roots and decomposing surface retained crop residues of previous crops with lower mineral nitrogen leading to lower priming effect. Whereas in CT system, due to availability of mineral nitrogen and other nutrients, microbial activity was higher in bulk soil and thereby facilitating more oxidation of SOM [17].

Higher soil moisture in bulk soils under CSA based systems irrespective of crop growth stages was due to crop residue retentions at soil surface (Suppl. Table 2). Crop residue mulch enhances soil water storage by regulating soil temperature, reducing evaporation and increasing infiltration and SOM concentration and thereby increasing water retention capacity of soil [9]. In bulk soils, SOC derived from crop residues interacts with soil matrix and enhances the specific surface area of soil which facilitates higher adsorption and retention of water molecules under CSA based systems [9].

For optimum plant growth and development, soil pH at which the ratio of enzymatic indicator (*AlP/AcP*) is about 0.5, can be considered as optimum [38]. In our experiment, both rhizosphere and bulk soil samples, the *AlP/AcP* value exceeded 0.5. These results were confirmed by soil pH measurement in soil:water 1:2 ratio (data not shown). Negative correlations between *AlP/AcP* and residue load, SOC and few enzymes might be due to the decline in soil pH because of release of organic acids upon decomposition of crop residues retained at soil surface [25]. The balance between three soil enzymes β-glucosidase, urease and phosphatase is quantified by alteration index three (*Al3*) which is sensitive to soil characteristics alterations. The low values of *Al3* indicated the better soil [26]. Higher values before sowing of crop irrespective of scenarios and sampling locations manifested poor soil quality which improved significantly upon growth of the plants as observed at maximum tillering and flowering stages. Importantly at harvest, significantly lower values of *Al3* were observed at bulk soils under CSA based systems which might be due to the higher SOC derived from crop residues decomposition. Higher SOC improves soil quality in CSA based systems and explains lower *Al3* values as evidenced from negative correlations between them (Table 2) [38]. Soil physico-chemical and biological properties are related to *GMea* of the studied enzymes and therefore, is used as an index of soil quality. Higher values of *GMea* designate better soil quality and can describe qualitative changes in soil without considering physicochemical properties [38]. Higher SOC in CSA based systems enhanced soil enzymes activities and subsequently *GMea* as evidenced from significant positive correlations between *GMea* with residue load, SOC and soil moisture content (Table 2). Lemanowicz et al. [38] also observed significant positive correlations between *GMea* and SOC while studying enzyme activities under different tree species in Poland.

Significant interactions among the scenarios, crop growth stages and rhizosphere and bulk soils on the soil enzymes might be due to the effect of Climate Smart Agriculture practices followed. Residue retention increases microbial population [39] by providing a stimulating substrate for their growth resulting in higher enzyme activities. Higher population counts of total bacteria, fluorescent Pseudomonas, and actinomycetes were observed under residue retention with ZT over residue removal under conventional tillage [40].

## 5 Conclusions

The enzyme activities are strongly influenced by tillage and crop establishment, crop rotation, and crop residues and water management practices. In bulk soil, enzymes activities were higher under CSA practices over conventional managements and activities of some of the enzymes were similar or comparable to rhizospheric soils. Rice based CSA systems showed higher enzyme activities over maize based systems. The CSA based systems has shown higher DHA and alkaline phosphatase activities before sowing rather than at maximum tillering, flowering and after harvesting of wheat. FDH activity in rice based CSA systems was 27% higher than maize based systems. Higher SOC was observed under CSA based systems which also influenced soil moisture availability due to crop residue retention. Crop management practices under a specific agro-ecosystem has important implications in nutrient availability to plants because upon decomposition, crop residues release nutrients which could help in savings of precious nutrients applied externally besides improving overall soil quality and carbon enrichment. Therefore, future studies should consider nutrients availability and priming effect at different crop growth stages in rhizosphere and bulk soils under CSA based cereal systems.

## Declaration of competing interest

The authors declare no competing interests.
