# Supplementary material for: Soil enzymes activity: Effect of climate smart agriculture on rhizosphere and bulk soil under cereal based systems of north-west India
Source: Eur J Soil Biol. 2021 Mar-Apr;103:103292. doi: 10.1016/j.ejsobi.2021.103292 (PMC7977442; doi:10.1016/j.ejsobi.2021.103292)
Supplement: Multimedia component 1 [file mmc1.doc]

Suppl.Table 1. Scenarios of agricultural change, crop rotation, tillage, crop establishment method, residue and nutrient management

| Scenario | Scenario description | Crop rotation | Tillage | Crop establishment method | Residue  management | Nutrient management  (NPK, kg/ ha) |
| --- | --- | --- | --- | --- | --- | --- |
| Sc1 | Farmer’s Practice | Rice-Wheat- Fallow | CT-CT | Rice: Transplanting  Wheat: Broadcasting | All residue removed | Rice: 175+58+0  Wheat: 150+58+0  (N through broadcasting) |
| Sc2 | Partial conservation agriculture based rice-wheat-mungbean system with flood irrigation (FI) | Rice-Wheat-Mungbean | CT-ZT-ZT | Rice: Transplanting  Wheat: Drill seeding  Mungbean: Drill/relay seeding | Full (100%) rice and anchored (25%) wheat residue retained on soil surface; full mungbean residue incorporated | Rice: 151+58+60  Wheat: 151+64+32  Mungbean: 0+0+0  (N through broadcasting) |
| Sc3 | Partial climate smart agriculture (CSA) based rice-wheat-mungbean system with FI | Rice-Wheat-Mungbean | ZT-ZT-ZT | Rice: Drill seeding  Wheat: Drill seeding  Mungbean: Drill/relay seeding | Full rice and mungbean; anchored wheat residue retained on soil surface | Rice: 162+64+62  Wheat: 151+64+32  Mungbean: 0+0+0  (N through broadcasting) |
| Sc4 | Partial CSA- based maize-wheat-mungbean system with FI | Maize-Wheat- Mungbean | ZT-ZT-ZT | Maize: Drill seeding  Wheat: Drill seeding  Mungbean: Drill/relay seeding | Partial (65%) maize and full mungbean; anchored wheat residue retained on soil surface | Maize: 174+64+62  Wheat: 151+64+32  Mungbean: 0+0+0  (N through broadcasting) |
| Sc5 | Full CSA- based rice-wheat-mungbean system with subsurface drip irrigation (Sc3+SDI) | Rice-Wheat-Mungbean | ZT-ZT-ZT | Same as in scenario 3 | Same as in scenario 3 | Rice: 130+64+62  Wheat: 121+64+32  Mungbean: 0+0+0  (N through Subsurface drip fertigation) |
| Sc6 | Full CSA- based maize-wheat-mungbean system with SDI (Sc5+SDI) | Maize-Wheat- Mungbean | ZT-ZT-ZT | Same as in scenario 4 | Same as in scenario 4 | Maize: 139+64+62  Wheat: 121+64+32  Mungbean: 0+0+0  (N through Subsurface drip fertigation) |

Where, CT-conventional tillage; ZT-zero tillage; FI: flood irrigation; SDI: subsurface drip irrigation; N-nitrogen; P-phosphorus; K-potassium

Time of rotation: Rice- June to October; Wheat- November to April, Mungbean- April to June and Maize- June to October

Suppl. Table 2. Total residue load (Mg ha^-1^) under different scenarios over the year

| Scenarios | 1^st^ year* | 2^nd^ year | 3^rd^ year | 4^th^ year | 5^th^ year | 6^th^ year | 7^th^ year | 8^th^ year | 9^th^ year | Total |
| --- | --- | --- | --- | --- | --- | --- | --- | --- | --- | --- |
| Sc1 | R/F* | R/F | R/F | R/F | R/F | R/F | R/F | R/F | R/F | -- |
| Sc2 | 10.3 | 11.4 | 12.3 | 13.8 | 15.4 | 15.3 | 10.5 | 10.9 | 15.7 | 115.5b |
| Sc3 | 15.2 | 17.4 | 11.8 | 11.7 | 10.8 | 9.6 | 9.3 | 10.3 | 14.6 | 110.6c |
| Sc4 | 15.6 | 20.2 | 14.0 | 16.0 | 14.7 | 16.3 | 8.8 | 10.0 | 13.3 | 129.0a |
| Sc5 | 15.2 | 17.4 | 11.8 | 11.7 | 10.8 | 9.6 | 9.7 | 10.6 | 14.8 | 111.6c |
| Sc6 | 15.6 | 20.2 | 14.0 | 16.0 | 14.7 | 16.3 | 8.4 | 10.4 | 13.3 | 129.0a |

Where: 1^st^ year: 2009-10, 2^nd^ year: 2010-11, 3^rd^ year: 2011-12, 4^th^ year: 2012-13, 5^th^ year: 2013-14, 6^th^ year: 2014-15, 7^th^ year: 2015-16, 8^th^ year: 2016-17, 9^th^ year: 2017-18

R-removed; F-fallow

Each value in each column is the sum of residues of rice, wheat and mungbean (Sc2, Sc3 and Sc5) and maize, wheat and mungbean (Sc4 and Sc6)

*Where,* Sc1-conventional rice-wheat system designated as conventional tillage system (CT system), Sc2- partial CA-based rice-wheat-mungbean system designated as partial CA based system (PCA-RW system), Sc3- Partial climate smart agriculture (CSA) based rice-wheat-mungbean system with flood irrigation (FI), Sc4- Partial CSA- based maize-wheat-mungbean system with FI, Sc5- Full CSA- based rice-wheat-mungbean system with subsurface drip irrigation (SDI), Sc6- Full CSA- based maize-wheat-mungbean system with SDI. Sc3 and Sc5 combinedly designated as rice based CSA system and Sc4 and Sc6 combinedly designated as maize based CSA system.

Same upper case letters are not significantly different at P < 0.05 according to Duncan Multiple Range Test (DMRT) for separation of mean

Suppl. Table 3. Acid phosphatase activity (µg p-NP g^-1^ soil hr^-1^) in rhizosphere and bulk soils under different tillage, residue and crop rotations

| Scenarios/  crop growth  stages | Rhizosphere | | | | Bulk soil | | | |
| --- | --- | --- | --- | --- | --- | --- | --- | --- |
|  | before sowing | maximum tillering | flowering | harvesting | before sowing | maximum tillering | flowering | harvesting |
| Sc1 | 77^BC^d | 91^A^cd | 114^C^a | 102^A^abc | 76^A^d | 94^C^bcd | 118^CD^a | 110^B^ab |
| Sc2 | 81^B^d | 126^A^a | 118^B^ab | 105^A^c | 89^A^d | 107^B^bc | 117^D^ab | 107^BC^bc |
| Sc3 | 82^B^e | 116^A^d | 100^D^d | 105^A^cd | 77^A^e | 123^A^ab | 100^E^d | 128^A^a |
| Sc4 | 69^C^e | 117^A^b | 119^B^b | 95^B^d | 67^A^e | 122^A^ab | 128^A^a | 102^CD^c |
| Sc5 | 76^BC^d | 105^A^cd | 133^A^bc | 104^A^ab | 91^A^a | 114^B^ab | 122^B^bc | 102^CD^bc |
| Sc6 | 100^A^bc | 114^A^ab | 114^C^ab | 94^B^c | 72^A^d | 107^B^a | 120^BC^ab | 101^D^bc |

*Where,* Sc1-conventional rice-wheat system designated as conventional tillage system (CT system), Sc2- partial CA-based rice-wheat-mungbean system designated as partial CA based system (PCA-RW system), Sc3- Partial climate smart agriculture (CSA) based rice-wheat-mungbean system with flood irrigation (FI), Sc4- Partial CSA- based maize-wheat-mungbean system with FI, Sc5- Full CSA- based rice-wheat-mungbean system with subsurface drip irrigation (SDI), Sc6- Full CSA- based maize-wheat-mungbean system with SDI. Sc3 and Sc5 combinedly designated as rice based CSA system and Sc4 and Sc6 combinedly designated as maize based CSA system.

Same upper case superscript letters among the scenarios and lower case letters among the crop growing stage irrespective of sampling places are not significantly different at P < 0.05 according to Duncan Multiple Range Test (DMRT) for separation of mean

Suppl. Table 4. Alkaline phosphatase activity (µg p-NP g^-1^ soil hr^-1^) in rhizosphere and bulk soils at different crop growth stages under different tillage, residue and crop rotations

| Scenarios/  crop growth  stages | Rhizosphere | | | | Bulk soil | | | |
| --- | --- | --- | --- | --- | --- | --- | --- | --- |
|  | before sowing | maximum tillering | flowering | harvesting | before sowing | maximum tillering | flowering | harvesting |
| Sc1 | 55^B^d | 121^A^ab | 112^B^abc | 98^AB^c | 61^B^d | 126^A^abc | 119^A^abc | 102^B^bc |
| Sc2 | 62^B^d | 136^A^a | 113^B^bc | 95^B^c | 66^B^d | 130^A^ab | 117^A^abc | 103^B^c |
| Sc3 | 70^B^d | 123^A^a | 116^A^a | 100^A^b | 85^B^c | 124^A^a | 114^B^a | 123^A^a |
| Sc4 | 35^C^c | 132^A^a | 88^D^b | 86^C^b | 80^B^b | 128^A^a | 93^C^b | 86^C^b |
| Sc5 | 103^A^b | 104^B^b | 98^C^bc | 88^C^cd | 133^A^a | 83^B^d | 90^C^bcd | 87^C^cd |
| Sc6 | 95^A^bc | 133^A^a | 85^D^cd | 88^C^cd | 99^AB^b | 127^A^a | 83^D^d | 87^C^cd |

*Where,* Sc1-conventional rice-wheat system designated as conventional tillage system (CT system), Sc2- partial CA-based rice-wheat-mungbean system designated as partial CA based system (PCA-RW system), Sc3- Partial climate smart agriculture (CSA) based rice-wheat-mungbean system with flood irrigation (FI), Sc4- Partial CSA- based maize-wheat-mungbean system with FI, Sc5- Full CSA- based rice-wheat-mungbean system with subsurface drip irrigation (SDI), Sc6- Full CSA- based maize-wheat-mungbean system with SDI. Sc3 and Sc5 combinedly designated as rice based CSA system and Sc4 and Sc6 combinedly designated as maize based CSA system.

Same upper case superscript letters among the scenarios and lower case letters among the crop growing stage irrespective of sampling places are not significantly different at P < 0.05 according to Duncan Multiple Range Test (DMRT) for separation of mean

Suppl. Table 5. Fluorescein diacetate hydrolases (FDH) activity (µg fluorescein g^-1^ soil hr^-1^) in rhizosphere and bulk soils at different crop growth stages under different tillage, residue and crop rotations

| Scenarios/  crop growth  stages | Rhizosphere | | | | Bulk soil | | | |
| --- | --- | --- | --- | --- | --- | --- | --- | --- |
|  | before sowing | maximum tillering | flowering | harvesting | before sowing | maximum tillering | flowering | harvesting |
| Sc1 | 44^C^ab | 54^A^a | 29^D^cd | 23^C^d | 37^B^bc | 54^AB^a | 29^B^cd | 49^A^ab |
| Sc2 | 51^B^a | 52^A^a | 45^AB^ab | 29^BC^c | 40^AB^b | 46^AB^ab | 39^A^b | 49^A^a |
| Sc3 | 55^A^ab | 50^A^b | 39^C^c | 38^A^c | 43^A^c | 60^A^a | 32^B^d | 53^A^b |
| Sc4 | 28^E^cd | 40^A^abc | 48^A^a | 33^AB^abc | 15^CD^d | 45^AB^ab | 30^B^bcd | 45^A^ab |
| Sc5 | 57^A^a | 39^A^b | 41^BC^b | 39^A^b | 18^C^c | 57^AB^a | 39^A^b | 41^A^b |
| Sc6 | 33^D^d | 44^A^ab | 49^A^a | 35^AB^cd | 1^D^e | 40^B^bc | 31^B^d | 44^A^ab |

*Where,* Sc1-conventional rice-wheat system designated as conventional tillage system (CT system), Sc2- partial CA-based rice-wheat-mungbean system designated as partial CA based system (PCA-RW system), Sc3- Partial climate smart agriculture (CSA) based rice-wheat-mungbean system with flood irrigation (FI), Sc4- Partial CSA- based maize-wheat-mungbean system with FI, Sc5- Full CSA- based rice-wheat-mungbean system with subsurface drip irrigation (SDI), Sc6- Full CSA- based maize-wheat-mungbean system with SDI. Sc3 and Sc5 combinedly designated as rice based CSA system and Sc4 and Sc6 combinedly designated as maize based CSA system.

Same upper case superscript letters among the scenarios and lower case letters among the crop growing stage irrespective of sampling places are not significantly different at P < 0.05 according to Duncan Multiple Range Test (DMRT) for separation of mean

Suppl. Table 6. Aryal Sulphatase activity (µg p-NP g^-1^ soil hr^-1^) in rhizosphere and bulk soils at different crop growth stages under different tillage, residue and crop rotations

| Scenarios/  crop growth  stages | Rhizosphere | | | | Bulk soil | | | |
| --- | --- | --- | --- | --- | --- | --- | --- | --- |
|  | before sowing | maximum tillering | flowering | harvesting | before sowing | maximum tillering | flowering | harvesting |
| Sc1 | 39^C^c | 18^C^e | 63^D^b | 76^CD^a | 30^C^cd | 29^A^d | 61^D^b | 74^B^a |
| Sc2 | 39^C^d | 57^A^c | 113^A^a | 77^BC^b | 41^AB^d | 39^A^d | 114^A^a | 76^B^b |
| Sc3 | 42^B^e | 35^B^e | 118^A^a | 78^B^d | 42^AB^e | 36^A^e | 109A^B^b | 87^A^c |
| Sc4 | 34^D^e | 47^A^d | 68^CD^bc | 74^D^ab | 38^B^e | 35^A^e | 64^D^c | 77^B^a |
| Sc5 | 45^A^bc | 30^B^c | 87^B^a | 78^B^a | 47^A^b | 39^A^bc | 86^C^a | 75^B^a |
| Sc6 | 42^B^d | 35^B^e | 74^C^c | 81^A^b | 37^BC^e | 28^A^f | 103^B^a | 80^B^b |

*Where,* Sc1-conventional rice-wheat system designated as conventional tillage system (CT system), Sc2- partial CA-based rice-wheat-mungbean system designated as partial CA based system (PCA-RW system), Sc3- Partial climate smart agriculture (CSA) based rice-wheat-mungbean system with flood irrigation (FI), Sc4- Partial CSA- based maize-wheat-mungbean system with FI, Sc5- Full CSA- based rice-wheat-mungbean system with subsurface drip irrigation (SDI), Sc6- Full CSA- based maize-wheat-mungbean system with SDI. Sc3 and Sc5 combinedly designated as rice based CSA system and Sc4 and Sc6 combinedly designated as maize based CSA system.

Same upper case superscript letters among the scenarios and lower case letters among the crop growing stage irrespective of sampling places are not significantly different at P < 0.05 according to Duncan Multiple Range Test (DMRT) for separation of mean

Suppl. Table 7. Urease activity (µg urea g^-1^ soil hr^-1^) in rhizosphere and bulk soils at different crop growth stages under different tillage, residue and crop rotations

| Scenarios/  crop growth  stages | Rhizosphere | | | | Bulk soil | | | |
| --- | --- | --- | --- | --- | --- | --- | --- | --- |
|  | before sowing | maximum tillering | flowering | harvesting | before sowing | maximum tillering | flowering | harvesting |
| Sc1 | 319^CD^b | 318^C^b | 319^B^b | 318^B^b | 316^AB^b | 335^A^a | 317^AB^b | 316^A^b |
| Sc2 | 321^C^b | 321^BC^b | 318^B^bcd | 319^AB^bc | 316^AB^cd | 341^A^a | 315^B^d | 316^A^cd |
| Sc3 | 317^DE^b | 333^AB^a | 316^B^b | 319^AB^b | 315^AB^b | 328^AB^a | 320^AB^b | 302^B^c |
| Sc4 | 326^B^ab | 314^C^cd | 330^A^a | 321^A^bc | 313^B^cd | 313^C^cd | 311^B^d | 321^A^abc |
| Sc5 | 315^E^ab | 326^BC^a | 319^B^ab | 319^AB^ab | 305^B^b | 317^BC^ab | 326^A^a | 319^A^ab |
| Sc6 | 331^A^b | 344^A^a | 324^AB^cde | 321^A^def | 328^A^bc | 316^BC^f | 325^A^bcd | 318^A^ef |

*Where,* Sc1-conventional rice-wheat system designated as conventional tillage system (CT system), Sc2- partial CA-based rice-wheat-mungbean system designated as partial CA based system (PCA-RW system), Sc3- Partial climate smart agriculture (CSA) based rice-wheat-mungbean system with flood irrigation (FI), Sc4- Partial CSA- based maize-wheat-mungbean system with FI, Sc5- Full CSA- based rice-wheat-mungbean system with subsurface drip irrigation (SDI), Sc6- Full CSA- based maize-wheat-mungbean system with SDI. Sc3 and Sc5 combinedly designated as rice based CSA system and Sc4 and Sc6 combinedly designated as maize based CSA system.

Same upper case superscript letters among the scenarios and lower case letters among the crop growing stage irrespective of sampling places are not significantly different at P < 0.05 according to Duncan Multiple Range Test (DMRT) for separation of mean

Suppl. Table 8. Cellulase activity (µg glucose g^-1^ soil hr^-1^) in rhizosphere and bulk soils at different crop growth stages under different tillage, residue and crop rotations

| Scenarios/  crop growth  stages | Rhizosphere | | | | Bulk soil | | | |
| --- | --- | --- | --- | --- | --- | --- | --- | --- |
|  | before sowing | maximum tillering | flowering | harvesting | before sowing | maximum tillering | flowering | harvesting |
| Sc1 | 0.41^C^g | 8.03^F^de | 12.59^D^ab | 8.54^B^cd | 4.29^D^f | 10.45^C^bc | 13.95^BC^a | 6.00^AB^ef |
| Sc2 | 0.95^B^f | 19.22^E^a | 17.97^A^b | 11.30^A^d | 6.86^D^e | 15.32^C^c | 19.12^A^a | 6.83^A^e |
| Sc3 | 0.92^B^e | 21.76^D^a | 17.39^AB^b | 7.99^B^d | 7.88^D^d | 15.39^C^bc | 13.56^BC^c | 6.52^A^d |
| Sc4 | 1.62^A^f | 35.63^C^a | 12.39^D^d | 7.16^B^e | 17.72^C^c | 27.99^B^b | 12.39^C^d | 4.53^B^ef |
| Sc5 | 1.77^A^f | 44.92^A^a | 15.34^C^d | 7.89^B^e | 29.83^A^c | 35.60^A^b | 16.33^AB^d | 7.06^A^ef |
| Sc6 | 1.60^A^f | 41.83^B^a | 15.95^BC^c | 7.31^B^e | 23.35^B^b | 39.79^A^a | 12.25^C^d | 5.37^AB^e |

*Where,* Sc1-conventional rice-wheat system designated as conventional tillage system (CT system), Sc2- partial CA-based rice-wheat-mungbean system designated as partial CA based system (PCA-RW system), Sc3- Partial climate smart agriculture (CSA) based rice-wheat-mungbean system with flood irrigation (FI), Sc4- Partial CSA- based maize-wheat-mungbean system with FI, Sc5- Full CSA- based rice-wheat-mungbean system with subsurface drip irrigation (SDI), Sc6- Full CSA- based maize-wheat-mungbean system with SDI. Sc3 and Sc5 combinedly designated as rice based CSA system and Sc4 and Sc6 combinedly designated as maize based CSA system.

Same upper case superscript letters among the scenarios and lower case letters among the crop growing stage irrespective of sampling places are not significantly different at P < 0.05 according to Duncan Multiple Range Test (DMRT) for separation of mean

Suppl. Table 9. Soil moisture content (%) in rhizosphere and bulk soils under different tillage, residue and crop rotations

| Scenarios | R-Before sowing | BS- Before sowing | R-Max tillering | BS -Max tillering | R-Flowering | BS -Flowering | R-At harvest | BS-At harvest |
| --- | --- | --- | --- | --- | --- | --- | --- | --- |
| Sc1 | 11.5c^A^ | 13.6c^A^ | 11.3c^A^ | 12.7c^A^ | 12.3c^A^ | 14.4c^A^ | 10.9b^B^ | 16.6bc^A^ |
| Sc2 | 11.2c^B^ | 14.3c^A^ | 20.5a^A^ | 19.4a^A^ | 13.3c^B^ | 16.7b^A^ | 15.0a^B^ | 18.8bc^A^ |
| Sc3 | 16.4a^A^ | 17.5b^A^ | 14.8bc^A^ | 13.4c^A^ | 16.3b^A^ | 11.4d^B^ | 14.8a^B^ | 39.0a^A^ |
| Sc4 | 15.0b^A^ | 16.5b^A^ | 17.1b^A^ | 18.9a^A^ | 14.1bc^B^ | 27.0a^A^ | 11.5b^B^ | 20.1b^A^ |
| Sc5 | 15.9a^B^ | 41.5a^A^ | 12.2c^A^ | 13.3c^A^ | 26.1a^A^ | 16.4b^B^ | 13.8ab^A^ | 13.5c^A^ |
| Sc6 | 15.6ab^A^ | 14.7c^A^ | 16.8b^A^ | 17.4b^A^ | 12.2c^A^ | 13.0cd^A^ | 14.8a^B^ | 17.8bc^A^ |

Where R: rhizosphere, BS: bulk soil

*Where,* Sc1-conventional rice-wheat system designated as conventional tillage system (CT system), Sc2- partial CA-based rice-wheat-mungbean system designated as partial CA based system (PCA-RW system), Sc3- Partial climate smart agriculture (CSA) based rice-wheat-mungbean system with flood irrigation (FI), Sc4- Partial CSA- based maize-wheat-mungbean system with FI, Sc5- Full CSA- based rice-wheat-mungbean system with subsurface drip irrigation (SDI), Sc6- Full CSA- based maize-wheat-mungbean system with SDI. Sc3 and Sc5 combinedly designated as rice based CSA system and Sc4 and Sc6 combinedly designated as maize based CSA system.

Similar lowercase letters among the scenarios and similar upper case superscript letters between rhizosphere and bulk soil are not significantly different at P < 0.05 according to Duncan Multiple Range Test (DMRT) for separation of mean.


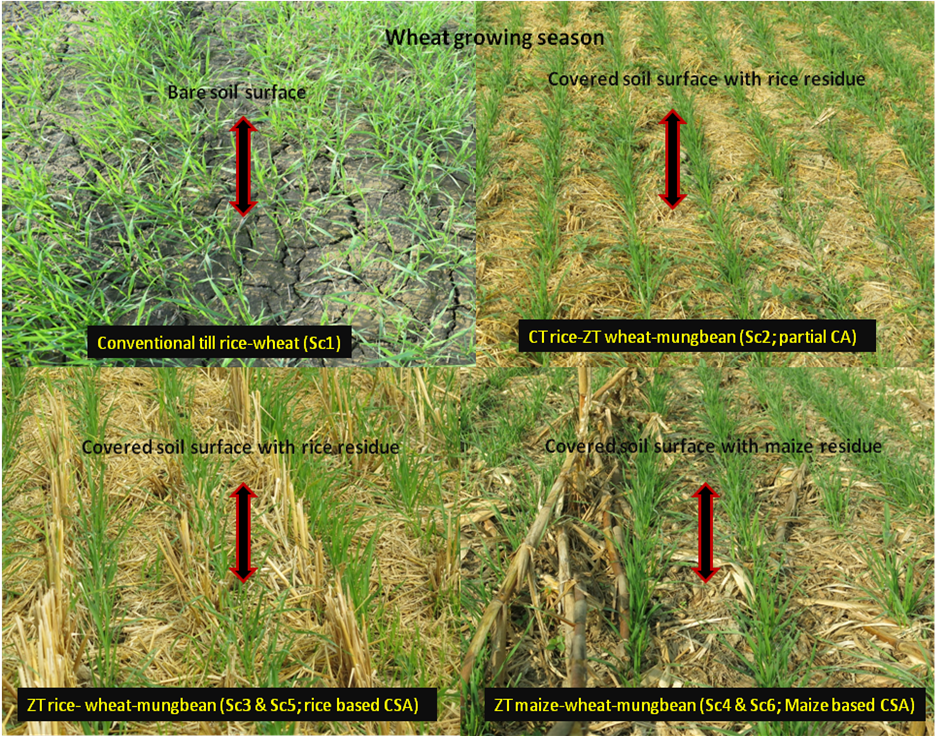


Suppl. Fig. 1. Different CSA practices showing bare soil surface in conventional agriculture and covered soil surface by rice and maize residues in CSA.

|  |  |
| --- | --- |
| Fig. 2a | Fig. 2b |
|  |  |
| Fig. 2c | Fig. 2d |

Suppl. Fig. 2. Beta-glucosidase activity (µg p-NP g^-1^ soil hr^-1^) in rhizosphere and bulk soils a) before sowing of crop, b) at maximum tillering c) flowering stage of crop and d) after harvesting of crop under different tillage, residue and crop rotations

Same upper case letters among the CSA systems and same lower case letters between rhizosphere and bulk soils in each system are not significantly different at P < 0.05 according to Duncan Multiple Range Test (DMRT) for separation of mean

Suppl. Fig. 3. Alkaline phosphatase (µg p-NP g^-1^ soil hr^-1^) and FDH (µg fluorescein g^-1^ soil hr^-1^) activity in rhizosphere and bulk soils after harvesting of crop under different tillage, residue and crop rotations

*Where*, R: rhizosphere; ALP: alkaline phosphatase activity

Same upper case letters among the CSA systems and same lower case letters between rhizosphere and bulk soils in each system are not significantly different at P < 0.05 according to Duncan Multiple Range Test (DMRT) for separation of mean
